# Substance use disorder among adolescents before and during the COVID-19 pandemic in Uganda: Retrospective findings from a psychiatric ward registry

**DOI:** 10.1371/journal.pone.0269044

**Published:** 2022-05-26

**Authors:** Mark Mohan Kaggwa, Joan Abaatyo, Emmanuel Alol, Moses Muwanguzi, Sarah Maria Najjuka, Alain Favina, Godfrey Zari Rukundo, Scholastic Ashaba, Mohammed A. Mamun

**Affiliations:** 1 Department of Psychiatry, Faculty of Medicine, Mbarara University of Science and Technology, Mbarara, Uganda; 2 African Centre for Suicide Prevention and Research, Mbarara, Uganda; 3 Faculty of Medicine, Mbarara University of Science and Technology, Mbarara, Uganda; 4 College of Health Sciences, Makerere University, Kampala, Uganda; 5 CHINTA Research Bangladesh, Savar, Dhaka, Bangladesh; 6 Department of Public Health, University of South Asia, Dhaka, Bangladesh; 7 Department of Public Health, Daffodil International University, Dhaka, Bangladesh; 8 Department of Public Health and Informatics, Jahangirnagar University, Savar, Dhaka, Bangladesh; Gulu University, UGANDA

## Abstract

It has been reported that the COVID-19 pandemic has predisposed adolescents to risky behaviors such as substance use and subsequent substance use disorder (SUD). However, it is unknown how the pandemic has changed the prevalence of SUD among adolescents in Uganda. We aimed to determine the prevalence of SUD and associated factors among adolescents in southwestern Uganda. Retrospectively, psychiatry ward records from November 2018 to July 2021 were collected from the largest tertiary hospital in southwestern Uganda. A total of 441 adolescent records were included in the analysis, with a mean age was 17±1.88 years, and the majority were males (50.34%). The overall prevalence of SUD was 7.26% (5.90% and 9.80% *before* and *during* the pandemic). Despite a little rise in SUD (3.9% increment) *during* the COVID-19 pandemic, there was no statistical difference compared to *before* the pandemic. The likelihood of being diagnosed with SUD was more among older adolescents at any period. In addition, having a diagnosis of bipolar mood disorder reduced the likelihood of SUD *during* the pandemic. This study indicated no statistical change in the diagnosis of SUD among adolescents before and *during* the COVID-19 pandemic. As older-male adolescents (17 to 19 years) were at higher risk of SUD, there is a need for early intervention for this group.

## Introduction

Adolescence (10–19 years) is characterized by a series of developmental changes, which are highly impacted by social, cultural, and nutritional influences [1]. A vast array of neurodevelopmental changes occur during this time, including cortical thinning, gray matter volume reductions, increases in white matter volume, synaptic pruning, and reorganization within cortical and limbic regions [2–5]. These neurodevelopmental changes give rise to characteristic behaviors during adolescence, such as improvements in cognition and executive functions, increases in reward sensitivity, novelty-seeking, risk-taking behavior, and a tendency to spend more time with peers [6–8]. Some of these behavioral characteristics, in turn, contribute to a greater likelihood of initiating substance use [9, 10].

Substance use by adolescents remains a significant public health concern [1]. More than 50% of substance use initiation cases occur during adolescence [11, 12]. The common substance used among adolescents is alcohol, followed by marijuana and cigarette smoking [13, 14]. In the USA, cigarette smoking trends have been increasing due to the introduction of e-cigarettes, although cannabis is the most common substance used by adolescents [14]. In sub-Sahara Africa–a region with the highest population of adolescents, the overall prevalence of adolescent substance use was estimated at 41.6% [15]. A study in Kenya among pregnant adolescents (aged 14–18 years) found alcohol use disorder at 13.2% [16]. In Uganda, 60–71% of school-going children (12 to 24 years) use addictive substances, especially alcohol (19.3%) and Kuber (smokeless tobacco, used sublingually) at 4.4% [13]. In a study among adolescents attending the Makerere/Mulago Columbia Adolescent Health Clinic in Mulago, 15.6% used at least an addictive substance, with alcohol being dominant at 15.2% of the total population [17]. Moreover, an earlier age of onset of substance use is significantly associated with the risk of developing a substance use disorder (SUD) later in life [1, 18, 19]. Despite studies showing a prevalence of substance use among adolescents in Uganda, no particular study has been carried out to investigate the prevalence of SUD in the country.

Prior Ugandan studies reported that factors associated with substance use (as opposed to substance use disorder) among adolescents include the death of a mother, suffering from chronic illness, depression, and having friends or family members with substance use problems [16, 17]. However, following the COVID-19 pandemic, substance use and substance use disorders have been reported to have increased along with changes in associated factors [20, 21]. The factors leading to the aforementioned increase in substance use are due to the drastic change in the lifestyle of most individuals due to lockdown, social isolation, restriction of movement, and economic shutdown [20, 21]. The COVID-19 associated social and economic restrictions led to decreased opportunities for regulation of stress, anxiety, existing mental health conditions, domestic violence, child maltreatment, and traumatic experiences, hence paving the way for substance use among adolescent victims as a mode of coping [20–23]. Eventually, suicide occurrences were caused by the inaccessibility of alcohol due to lockdown, and alcohol withdrawal symptoms were reported during the pandemic [24, 25].

In Uganda, there is limited evidence on the impact of the COVID-19 pandemic on substance use disorder among the vast young population. Yet, the country had its schools closed for almost two years with no online studies or other means [26], thus removing this known protective factor (staying in school and school connectedness) against substance use among adolescents [27]. Furthermore, previous literature reports that adolescents out of school are at a higher risk of using addictive substances [28], thus putting them at risk of being diagnosed with SUD. Therefore, in this study, a retrospective record review was performed to compare the prevalence and associated factors of SUD based on two-time points, that is, *before* and *during* the COVID-19 pandemic among adolescents with mental health problems attending a psychiatric ward in Uganda.

## Methods

### Study area and design

This study was based on a retrospective review of the outpatient registers (Health Management Information Systems [HMIS] form 031) of the Mbarara Regional Referral Hospital (MRRH) psychiatry ward from November 2018 to July 2021. The psychiatry unit manages patients with mental illnesses confirmed by mental health professionals only and those with medical conditions that have mental illness presentations such as HIV. MRRH psychiatry ward is the largest psychiatric facility in southwestern Uganda, with a stationed child and adolescent psychiatrist and a functional specialist addiction clinic. Therefore, the facility handles and manages many adolescents especially referrals from all over southwestern Uganda, with mental illnesses, including SUD. *HMIS form 031* captures the following information; patient’s number, name, address, age, anthropometric measurements, gender, next of kin, substances of addiction used, investigations, physical symptoms, diagnosis, and treatment given. At the beginning of the COVID-19 pandemic in Uganda (March 2020), 16 months data of *before* the COVID-19 pandemic and 17 months *during* the pandemic, were collected for this study.

### Inclusion and exclusion criteria

Using the *HMIS form 031* register, information of all adolescents aged 10 to 19 years [29] was extracted. After retrieving all the eligible registers from the psychiatry department records office, a total of 720 records were retrieved. Due to reattendances to the clinic, records with the same patient number, age, gender, patients’ names initials, year of attendance, and diagnosis were considered duplicates, and only one record was included in this study. A total of 279 records were reattendances in the same calendar year for the same diagnosis, and so were excluded from the final analysis as duplicates.

### Data collection, management and quality control

The following information was extracted from the *HMIS form 031*: (i) patient’s number, (ii) patient name initials, (iii) age, (iv) gender (male, female), (v) substances of addiction used (alcohol; cannabis; cigarettes (smoked tobacco), Kuber (smokeless tobacco, commonly used sublingually) [30]; miraa/khat [*Catha Edulis Forsk*]–plant leaves that contain stimulant similar to Amphetamine [31]; others such as Akandi [32], and glue), and (vi) diagnosis. Age was categorized into three groups based on the stage of adolescence [33]; early adolescents (10–14 years), middle adolescence (15–17 years), and late adolescence (18 to 19 years). Data were entered parallel by two pairs of individuals (JA and EA) and (SMN and CK), and in case of any discrepancies, MMK resolved them. Data were cleaned in Microsoft Excel.

### Substance use disorder and mental health diagnosis

The mental health diagnoses at the MRRH psychiatry ward are based on the ICD-11. Patients with physical diagnoses are referred to the department for management of their psychiatric manifestations or comorbidities. For this study, a diagnosis of SUD was considered for an individual who had been diagnosed with any type of SUD, such as alcohol use disorder, cannabis use disorder, among others [34]. An individual can be diagnosed with more than one type of substance use disorder. In addition, individuals with SUD can have both mental comorbidities such as depression, anxiety, or physical comorbidities such as HIV.

### Ethics

The present study was conducted in accordance with the Declaration of Helsinki 2013 [35] and was approved by the Mbarara University of Science and Technology research ethics committee (MUST-2021-229). However, the formal consent was waivered since the data was retrospective, and it was not possible to track the involved participants. All of the information is anonymously presented in this study and there are no ethical concerns.

### Data analysis

Data were analyzed using STATA version 12.0. Categorical variables were presented with frequencies and percentages. Age was presented in terms of mean and standard deviation. The Gaussian assumption was used to assess for normality based on the Shapiro-Wilks test and histograms. Chi-square tests for categorical variables or student *t*-tests for continuous variables were performed to determine significant differences between individuals with a diagnosis of SUD and those without. Inferential analysis of two groups, *before* and *during* the pandemic, was performed with the included study variables. Binary logistic analysis for factors associated with SUD was performed. Factors significant at bivariate logistic analysis were tested for collinearity using variance inflation factors (VIF), and those with a VIF below three were included in the final model at multiple logistic regression. The significant level was at less than 5% for a 95% confidence interval.

## Results

A total of 441 records were finally included in the present analysis, with 65.31% (n = 288) being recorded *before* the COVID-19 pandemic. The average age of the participants included was 17±1.88 years, with almost half (48.75%) being in their late adolescence (18–19 years). About half were males (50.34%), and the commonest psychiatric diagnosis made was bipolar disorder (40.59%), followed by schizophrenia and other primary psychosis (27.66%). Detailed information on adolescent data use in this study can be found in **Table 1**.

**Table 1:** Distribution of the socio-demographics and comorbid mental and physical illnesses with substance abuse disorder.

Variable	Total; n (%)	Substance use disorder		X^2^ (p-value)
		No; 409 (92.74%)	Yes; 32 (7.26%)	
**Socio-demographic information**				
**Admission time**				
Before pandemic	288 (65.31)	271 (94.10)	17 (5.90)	2.26 (0.133)
During pandemic	153 (34.69)	138 (90.20)	15 (9.80)	
**Age (mean, *SD)***	17.00,1.88	16.91,1.90	18.16,1.02	**-3.66 (<0.001)**
**Stage of adolescence**				
Early	43 (9.75)	43 (100)	0	**17.91 (<0.001)**
Middle	183 (41.50)	178 (97.27)	5 (2.73)	
Late	215 (48.75)	188 (87.44)	27 (12.56)	
**Gender**				
Female	217 (49.66)	217 (100)	0	**34.06 (<0.001)**
Male	220 (50.34)	188 (85.45)	32 (14.55)	
**Comorbid mental illnesses and physical illnesses**				
**1. Schizophrenia and other primary psychosis (6A2)**				
No	319 (72.34)	288 (90.28)	31 (9.72)	**10.38 (<0.001)**
Yes	122 (27.66)	121 (99.18)	1 (0.82)	
**2. Bipolar mood disorders (6A8)**				
No	262 (59.41)	232 (88.55)	30 (11.45)	**16.87 (<0.001)**
Yes	179 (40.59)	177 (98.88)	2 (1.12)	
**3. Depression**				
No	394 (89.34)	363 (92.13)	31 (7.87)	0.36 (0.548)
Yes	47 (10.66)	46 (97.87)	1 (2.13)	
**4. Anxiety and fear-related disorders (6B0)**				
No	426 (96.60)	394 (92.49)	32 (7.51)	1.21 (0.270)
Yes	15 (3.40)	(100)	0	
**5. Neurodevelopmental disorders (6A0)**				
No	431 (97.73)	399 (92.58)	32 (7.42)	0.80 (0.371)
Yes	10 (2.27)	10 (100)	0	
**6. Factitious disorders (6D5)**				
No	435 (98.64)	403 (92.64)	32 (7.36)	0.48 (0.490)
Yes	6 (1.36)	6 (100)	0	
**7. Elimination disorders (6C0)**				
No	440	408 (92.73)	32 (7.27)	0.08 (0.779)
Yes	1	1 (100)	0	
**8. Personality disorder (8D1)**				
No	439 (99.55)	407 (92.71)	32 (7.29)	0.16 (0.692)
Yes	2 (0.45)	2 (100)	0	
**9. Dissociative disorders (6B6**)				
No	440 (99.77)	408 (92.73)	32 (7.27)	0.08 (0.779)
Yes	1 (0.23)	1 (100)	0	
**10. Disruptive behavior and dissocial disorders (6C9)**				
No	439 (99.55)	407 (92.71)	32 (7.29)	0.16 (0.692)
Yes	2 (0.45)	2 (100)	0	
**11. Catatonia**				
No	440 (99.77)	408 (92.73)	32 (7.27)	0.08 (0.779)
Yes	1 (0.23)	1 (100)	0	
**12. Disorders specifically associated with stress disorders (6B4)**				
No	431 (97.73)	400 (92.81)	31 (7.19)	0.11 (0.735)
Yes	10 (2.27)	9 (90.00)	1 (10.00)	
**13. Mental and behavioral disorders associated with pregnancy, childbirth and the puerperium, (6E2)**				
No	440 (99.77)	408 (92.73)	32 (7.27)	0.08 (0.779)
Yes	1 (0.23)	1 (100)	0	
**14. Disorders of bodily distress and bodily experience (6C2)**				
No	432 (97.96)	400 (92.59)	32 (7.41)	0.72 (0.397)
Yes	9 (2.04)	9 (100)	0	
**15. Impulsive control disorders (6C7)**				
No	438 (99.32)	406 (92.69)	32 (7.31)	0.24 (0.627)
Yes	3 (0.68)	3 (100)	0	
**16. HIV**				
No	428 (97.05)	396 (92.52)	32 (7.48)	1.05 (0.306)
Yes	13 (2.95)	13 (100)	0	

### Prevalence of substance abuse disorder and substance use types

**Fig 1:**
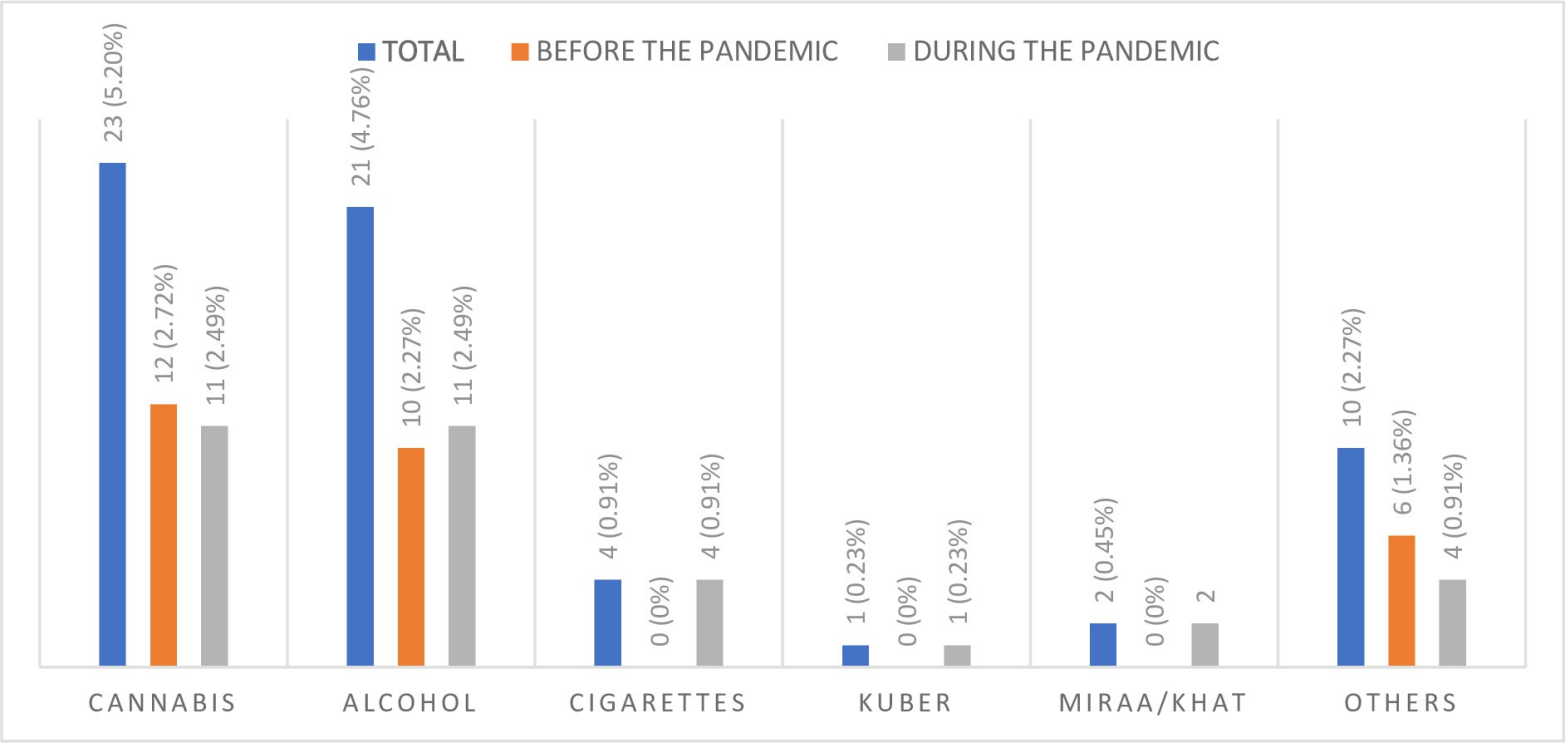
Substances used *before* and *during* the COVID-19 pandemic.

The overall prevalence of SUD was 7.26% (n = 32), where 5.90% (n = 17) adolescents had SUD *before* the pandemic and 9.80% (n = 15) *during* the pandemic. Although a 3.9% increment of SUD *during* the COVID-19 pandemic was observed, the difference was not statistically significant (χ^2^ = 2.26, *p* = 0.133). The most used substance was cannabis (5.21%, n = 23/441), followed by alcohol, 4.76%, (n = 21/441), and the least used was Kuber 0.23% (n = 1/441). There was a statistically significant difference between cigarette use and the pandemic period (p = 0.023), although other substances were not significantly associated (**Fig 1**).

### Distribution of study variables with substance use disorder

The mean age of adolescents with SUD was higher compared to those without SUD (18.16 vs. 16.91 years; t = -3.66, *p*<0.001), that is, 12.56% of late adolescents had been diagnosed with SUD, whereas it was 2.73% for middle-aged adolescents and there was no SUD case in early adolescents, and this association of age group with SUD was statistically significant χ^2^ = 17.91, *p*<0.001). Furthermore, all adolescents with SUD were males (14.55% vs. 0%), and the association between gender and SUD was statistically significant (χ^2^ = 34.06, *p*<0.001). In addition, adolescents with schizophrenia and bipolar mood disorders were less likely to be diagnosed with SUD. For details about the relationship between the study variables and SUD, please refer to **Table 1**.

There was a statistical difference between the stage of adolescents and SUD *before* the COVID-19 pandemic (χ^2^ = 14.77, *p* = 0.001), whereas no difference was observed *during* the pandemic. More males were statistically more diagnosed with SUD during the two periods (*p*<0.001); however, they were more prevalent *during* the pandemic than *before* the pandemic (18.29% vs. 12.32%). *Before* the pandemic, adolescents without comorbidity of schizophrenia and other primary psychosis (6A2) were less prone to SUD (8.13% vs. 0%, χ^2^ = 6.83, *p* = 0.009); but such association was not statistically significant *during* the pandemic. Similarly, adolescents without comorbid bipolar disorder were more likely to be diagnosed with SUD in both periods (**Table 2**).

**Table 2:** Distribution of the socio-demographics and comorbid mental and physical illnesses with substance abuse disorder based on *before* and *during* the pandemic.

Variables	Before COVID-19 pandemic			During the COVID-19 pandemic		
	No	Yes	χ^2^ (*p-*value)	No	Yes	χ^2^ (*p-*value)
	(271, 94.10%)	(17, 5.90%)		(138, 90.20%)	(15, 9.80%)	
**Stage of adolescence**						
Early	29 (100)	0	**14.77 (0.001)**	14 (100)	0	4.64 (0.098)
Middle	117 (99.15)	1 (0.85)		61 (93.85)	4 (6.15)	
Late	125 (88.65)	16 (11.35)		63 (85.14)	11 (14.86)	
**Gender**						
Female	148 (100)	0	**19.38 (<0.001)**	69 (100)	0	**14.01 (<0.001**)
Male	121 (87.68)	17 (12.32)		67 (81.71)	15 (18.29)	
**Comorbid mental illnesses and physical illnesses**						
**1. Schizophrenia and other primary psychosis (6A2)**						
No	192 (91.87)	17 (8.13)	**6.83 (0.009)**	96 (87.27)	14 (12.73)	3.78 (0.052)
Yes	79 (100)	0		42 (97.67)	1 (2.33)	
**2. Bipolar mood disorders (6A8)**						
No	156 (90.17)	17 (9.83)	**12.01 (0.001)**	76 (85.39)	13 (14.61)	**5.55 (0.018)**
Yes	115 (100)	0		62 (96.88)	2 (3.13)	
**3. Depression**						
No	239 (93.36)	17 (6.64)	2.26 (0.133)	124 (89.86)	14 (10.14)	
Yes	32 (100)	0		14 (93.33)	1 (6.67)	0.18 (0.667)
**4. Anxiety and fear-related disorders (6B0)**						
No	262 (93.91)	17 (6.09)	0.58 (0.445)	132 (89.80)	15 (10.20)	0.68 (0.410)
Yes	9 (100)	0		6 (100)	0	
**5. Neurodevelopmental disorders (6A0)**						
No	264 (93.95)	17 (6.05)	0.45 (0.502)	135 (90.00)	15 (10.00)	0.33 (0.564)
Yes	7 (100)	0		3 (100)	0	
**6. Factitious disorders (6D5)**						
No	266 (93.99)	17 (6.01)	0.32 (0.572)	137 (90.13)	15 (9.87)	0.11 (0.741)
Yes	5 (100)	0		1 (100)	0	
**7. Elimination disorders (6C0)**						
No	270 (94.08)	17 (5.92)	0.06 (0.802)	138 (90.20)	15 (9.80)	NA
Yes	1 (100)	0		0	0	
**8. Personality disorder (8D1)**						
No	269 (94.06)	17 (5.94)	0.13 (0.722)	138 (90.20)	15 (9.80)	NA
Yes	2 (100)	0		0	0	
**9. Dissociative disorders (6B6**)						
No	270 (94.08)	17 (5.92)	0.06 (0.802)	138 (90.20)	15 (9.80)	NA
Yes	1 (100)	0		0	0	
**10. Disruptive behavior and dissocial disorders (6C9)**						
No	270 (94.08)	17 (5.92)	0.06 (0.802)	137 (90.13)	15 (9.87)	0.11 (0.741)
Yes	1 (100)	0		1 (100)	0	
**11. Catatonia**						
No	271 (94.10)	17 (5.90)	NA	137 (90.13)	15 (9.87)	0.11 (0.741)
Yes	0	0		1 (100)	0	
**12. Disorders specifically associated with stress disorders (6B4)**						
No	267 (94.01)	17 (5.99)	0.25 (0.614)	133 (90.48)	14 (9.52)	0.33 (0.564)
Yes	4 (100)	0		5 (83.33)	1 (16.67)	
**13. Mental and behavioral disorders associated with pregnancy, childbirth and the puerperium, (6E2)**						
No	271 (94.10)	17 (5.90)	NA	137 (90.13)	15 (9.87)	0.11 (0.741)
Yes	0	0		1 (100)	0	
**14. Disorders of bodily distress and bodily experience (6C2)**						
No	264 (93.95)	17 (6.05)	0.45 (0.502)	136 (90.07)	15 (9.93)	0.22 (0.639)
Yes	7 (100)	0		2 (100)	0	
**15. Impulsive control disorders (6C7)**						
No	269 (94.06)	17 (5.94)	0.13 (0.722)	136 (90.07)	15 (9.93)	0.22 (0.639)
Yes	2 (100)	0		2 (100)	0	
**16. HIV**						
No	261 (93.88)	17 (6.12)	0.65 (0.420)	135 (90.00)	15 (10.00)	0.33 (0.564)
Yes	10 (100)	0		3 (100)	0	

### Factors associated with having a substance use disorder diagnosis

The variables that were significant (*p*<0.05) at inferential statistics (i.e., chi-square and t-test), were included in logistic analyses and those significant at bivariate logistic regressions were considered for model building. For both periods combined, all the included variables; age, comorbidity of schizophrenia and other primary psychosis (6A2), and bipolar disorder had a VIF below 3 and mean VIF of 1.21, indicating good collinearity. The final model had a sensitivity of 0%, specificity of 100%, a negative predictive value of 92.74%, and would correctly classify 92.74% of the diagnosis of SUD in both periods. The model had good goodness of fit for all the included variables, with a *p*-value of 0.590. The likelihood of being diagnosed with SUD increased with age [adjusted odds ratio (aOR) = 1.81, 95% confidence interval (CI) = 1.31–2.52, *p*<0.001]. However, having a diagnosis of bipolar mood disorder (aOR = 0.04, CI = 0.01–0.22, *p* = 0.001) and schizophrenia and other primary psychosis (aOR = 0.03, CI = 0.01–0.13, *p*<0.001) was protective against SUD.

*Before* the pandemic, only an increase in age was associated with SUD; whereas an increase in age and having comorbidity of bipolar disorder were associated with SUD *during* the pandemic. These were tested for collinearity and all had a VIF of 1. Therefore, they were included in the final model, which had a sensitivity of 0%, specificity of 100%, a negative predictive value of 90.20%, and would correctly classify 90.20% of the diagnosis of SUD *during* the pandemic. The likelihood of being diagnosed with SUD increased with an increase in age *during* the pandemic (aOR = 1.72, CI = 1.06–2.80, *p* = 0.028). However, having a diagnosis of bipolar disorder reduced the likelihood of being diagnosed with SUD (aOR = 0.18, CI = 0.04–0.87, *p* = 0.032) (**Table 3**).

**Table 3:** Logistic analysis for factors associated with substance use disorder.

Variable	Total sample				Before the pandemic		During the pandemic			
	Bivariable analysis		Multivariable analysis		Bivariable analysis		Bivariable analysis		Multivariable analysis	
	Crude Odds ratio (95% confidence interval)	p-value	Crude Odds ratio (95% confidence interval)	p-value	Crude Odds ratio (95% confidence interval)	p-value	Crude Odds ratio (95% confidence interval)	p-value	Adjusted odds ratio (95% confidence interval)	P-value
**Age**	1.86 (1.31–2.62)	**<0.001**	1.81 (1.31–2.52)	**<0.001**	1.98 (1.20–3.24)	**0.007**	1.76 (1.08–2.86)	**0.023**	1.72 (1.06–2.80)	**0.028**
**Gender**										
Female	1				1		1			
Male	Omitted				Omitted		Omitted			
**Schizophrenia and other primary psychosis (6A2)**										
No	1		1		1		1			
Yes	0.77 (0.01–0.57)	**0.012**	0.03 (0.01–0.22)	**0.001**	Omitted		0.16 (0.02–1.28)	0.085		
**Bipolar mood disorders**										
No	1		1		1		1		1	
Yes	0.09 (0.02–0.37)	**0.001**	0.04 (0.01–0.17)	**<0.001**	Omitted		0.19 (0.04–0.87)	**0.032**	0.18 (0.04–0.87)	**0.032**

## Discussion

This study aimed to investigate substance use disorder (SUD) among adolescents *before* and *during* the COVID-19 pandemic using a retrospective record review of the outpatient register (HMIS form 031 at the Mbarara Regional Referral psychiatry ward, the largest tertiary hospital in southwestern Uganda). The overall prevalence of SUD was 7.26% considering all the adolescent records over two periods (5.90% and 9.80%, *before* and *after* the pandemic, respectively). There was no statistically significant difference in SUD during the two periods despite a slightly higher rate reported *during* the pandemic. In addition, the use of cigarettes was statistically higher *during* the COVID-19 pandemic. The likelihood of being diagnosed with SUD was more among older adolescents at any period. However, having a diagnosis of bipolar mood disorder reduced the likelihood of being diagnosed with SUD *during* the pandemic.

Despite a little rising in SUD (3.9% increase) *during* the COVID-19 pandemic, there was no statistically significant change compared to *before* the pandemic; a finding similar to other studies that found that cannabis and alcohol use did not change during the pandemic [36]. Lockdowns during the pandemic presented an opportunity to reduce the availability of substances due to the disruption in the distribution chain, as seen in other countries. For example, a 17% and 24% decline in marijuana and alcohol availability in the USA was reported during the pandemic; given the situation of substance unavailability in the USA, the substance use rates had not significantly decreased [36]. Despite these findings, other studies reported increased substance use among adolescents during the pandemic of 25.33% and 9.33% increment in alcohol and other substance use, respectively [37].

The use of substances in our study was higher than that reported in Indonesia at 5.3% for the commonly used substance—alcohol [38]. Cigarette use was statistically significantly more during the COVID-19 pandemic, which is contradictory to other studies that found no significant comparison [36]. These findings indicate that adolescents procured and accessed substances from their own homes since schools were closed during the pandemic [39]. Vaping materials and cigarettes are generally obtained through peer networks [40], and their availability kept increasing due to increased interaction during school closure making cigarettes more accessible during the lockdown.

This study found that an increase in age was associated with an increased likelihood of being diagnosed with SUD, a factor consistently associated with substance use among adolescents, as reported by many researchers [38, 40]. An increase in age leads to increased curiosity, bandwagon effect, and impulsivity towards substance use [41]. The use of addictive substances, particularly alcohol, is restricted under 18 in Uganda [42]. Therefore, adolescents who tend towards the accepted age (late adolescence, 18 to 19 years in this study; for example) were more likely to be diagnosed with SUD.

Males are more likely than females to take alcohol and drugs. The more exposure to a substance, the higher the risk of being diagnosed with SUD. Male’s predominance in substance use may be associated with the cultural acceptance of substance use among the male gender, especially in this patriarchal society [43, 44]. Also, the use of substances shows the masculinity in an individual man since weak men are believed to be unable to handle alcohol or other substances [44, 45]. Moreover, adolescents are always watching television and on the internet, which has adverts promoting male use of substances. For example, the use of alcohol by most celebrities in songs and most sports are sponsored by alcohol-producing companies [28]. The increased likelihood of being diagnosed with SUD among males could also be an effect of the increased tolerance towards alcohol that puts them at a higher risk for SUD [45, 46].

Individuals with primary psychotic or mood disorders usually use addictive substances to cope with their symptoms [47, 48]. Although teenagers with primary psychosis or mood symptoms mostly experience their first episodes of the primary disorder, most of them have not yet developed coping mechanisms, including substance use, unlike their counterparts who have experienced several episodes. This may be the case supporting the present finding that having a diagnosis of a primary psychotic or mood disorder was less associated with SUD.

The interpretation of this study’s findings should be made with caution in view of the following limitations. First of all, it was a retrospective cross-sectional study whose data depended on the efficiency of record reporting by the department staff, an aspect that has caused inconsistencies with HMIS reporting [49]. In addition, many variables that could have influenced the findings, such as stressors and family environment, were not retrieved from the registry and hence not included in the analysis. Finally, the data included in the analysis were from one facility, limiting the generalization of the findings throughout the country.

## Conclusions

The present study is one of the first approaches to investigating the change of SUD after the COVID-19 pandemic inception. The findings reported herein indicate a slight increment of SUD among Ugandan adolescents during the pandemic. Therefore, there is a need to screen and treat adolescents for SUD as the pandemic progresses, especially among male-older adolescents. Some strategies, for example, reopening schools, and increasing support in schools and families for managing and identifying SUD to contribute towards early intervention for preventing the hazardous consequences of SUD, such as an increase in crime, are highly suggested.

## References

[1] Hamidullah S, Thorpe HHA, Frie JA, Mccurdy RD, Khokhar JY. Adolescent substance use and the brain: behavioral, cognitive and neuroimaging correlates. *Frontiers in Human Neuroscience*. 2020;14(298). doi: 10.3389/fnhum.2020.00298 32848673 PMC7418456

[2] Schneider M. Adolescence as a vulnerable period to alter rodent behavior. *Cell and Tissue Research*. 2013;354(1):99–106. Epub 2013/02/23. doi: 10.1007/s00441-013-1581-2 23430475

[3] Jaworska N, MacQueen G. Adolescence as a unique developmental period. *Journal of Psychiatry & Neuroscience*: JPN. 2015;40(5):291–3. Epub 2015/08/21. doi: 10.1503/jpn.150268 26290063 PMC4543091

[4] Dumontheil I. Adolescent brain development. *Current Opinion in Behavioral Sciences*. 2016;10:39–44. doi: 10.1016/j.cobeha.2016.04.012

[5] Thorpe HHA, Hamidullah S, Jenkins BW, Khokhar JY. Adolescent neurodevelopment and substance use: receptor expression and behavioral consequences. *Pharmacology & Therapeutics*. 2020;206:107431. Epub 2019/11/11. doi: 10.1016/j.pharmthera.2019.107431 31706976

[6] Spear LP. The adolescent brain and age-related behavioral manifestations. *Neuroscience and Biobehavioral Reviews*. 2000;24(4):417–63. Epub 2000/05/19. doi: 10.1016/s0149-7634(00)00014-2 10817843

[7] Choudhury S, Blakemore SJ, Charman T. Social cognitive development during adolescence. *Social Cognitive and Affective Neuroscience*. 2006;1(3):165–74. Epub 2008/11/06. doi: 10.1093/scan/nsl024 18985103 PMC2555426

[8] Romer D. Adolescent risk taking, impulsivity, and brain development: implications for prevention. *Developmental Psychobiology*. 2010;52(3):263–76. Epub 2010/02/23. doi: 10.1002/dev.20442 20175097 PMC3445337

[9] Lisdahl KM, Sher KJ, Conway KP, Gonzalez R, Feldstein Ewing SW, Nixon SJ, et al. Adolescent brain cognitive development (ABCD) study: overview of substance use assessment methods. *Developmental Cognitive Neuroscience*. 2018;32:80–96. Epub 2018/03/22. doi: 10.1016/j.dcn.2018.02.007 29559216 PMC6375310

[10] Garofoli M. Adolescent substance abuse. *Primary Care*. 2020;47(2):383–94. Epub 2020/05/20. doi: 10.1016/j.pop.2020.02.013 32423721

[11] Blanco C, Flórez-Salamanca L, Secades-Villa R, Wang S, Hasin DS. Predictors of initiation of nicotine, alcohol, cannabis, and cocaine use: results of the National Epidemiologic Survey on Alcohol and Related Conditions (NESARC). *The American Journal on Addictions*. 2018;27(6):477–84. Epub 2018/08/09. doi: 10.1111/ajad.12764 30088294

[12] Gray KM, Squeglia LM. Research Review: What have we learned about adolescent substance use? *Journal of Child Psychology and Psychiatry*. 2018;59(6):618–27. Epub 2017/07/17. doi: 10.1111/jcpp.12783 28714184 PMC5771977

[13] Abbo C, Okello ES, Muhwezi W, Akello G, Ovuga E. Alcohol, substance use and psychosocial competence of adolescents in selected secondary schools in Uganda: a cross sectional survey. *International Neuropsychiatric Disease Journal*. 2016;7(2):25387. doi: 10.9734/INDJ/2016/25387 27398388 PMC4936516

[14] Johnston LD, Miech RA, O’Malley PM, Bachman JG, Schulenberg JE, Patrick ME. Monitoring the Future National Survey Results on Drug Use, 1975–2017: Overview, key findings on adolescent drug use. Institute for Social Research, University of Michigan, 2018.Available from: http://files.eric.ed.gov/fulltext/ED594190.pdf.

[15] Olawole-Isaac A, Ogundipe O, Amoo EO, Adeloye D. Substance use among adolescents in sub-Saharan Africa: a systematic review and meta-analysis. *South African Journal of Child Health*. 2018;2018(1). Available from: https://hdl.handle.net/10520/EJC-10deb396ce

[16] Kimbui E, Kuria M, Yator O, Kumar M. A cross-sectional study of depression with comorbid substance use dependency in pregnant adolescents from an informal settlement of Nairobi: drawing implications for treatment and prevention work. *Annals of General Psychiatry*. 2018;17(1):53. doi: 10.1186/s12991-018-0222-2 30598688 PMC6300883

[17] Henry MB, Bakeera-Kitaka S, Lubega K, Snyder SA, LaRussa P, Pfeffer B. Depressive symptoms, sexual activity, and substance use among adolescents in Kampala, Uganda. *African Health Sciences*. 2019;19(2):1888–96. doi: 10.4314/ahs.v19i2.12 . 31656472 PMC6794506

[18] Taioli E, Wynder EL. Effect of the age at which smoking begins on frequency of smoking in adulthood. *The New England Journal of Medicine*. 1991;325(13):968–9. Epub 1991/09/26. doi: 10.1056/NEJM199109263251318 1881424

[19] Viner RM, Taylor B. Adult outcomes of binge drinking in adolescence: findings from a UK national birth cohort. *Journal of Epidemiology and Community Health*. 2007;61(10):902–7. Epub 2007/09/18. doi: 10.1136/jech.2005.038117 17873228 PMC2652971

[20] Enns A, Pinto A, Venugopal J, Grywacheski V, Gheorghe M, Kakkar T, et al. Substance use and related harms in the context of COVID-19: a conceptual model. *Health Promotion and Chronic Disease Prevention in Canada*: *Research*, *Policy and Practice*. 2020;40(11–12):342–9. Epub 2020/09/17. doi: 10.24095/hpcdp.40.11/12.03 32936071 PMC7745829

[21] Wang QQ, Kaelber DC, Xu R, Volkow ND. COVID-19 risk and outcomes in patients with substance use disorders: analyses from electronic health records in the United States. *Molecular Psychiatry*. 2021;26(1):30–9. Epub 2020/09/16. doi: 10.1038/s41380-020-00880-7 32929211 PMC7488216

[22] Fegert JM, Vitiello B, Plener PL, Clemens V. Challenges and burden of the Coronavirus 2019 (COVID-19) pandemic for child and adolescent mental health: a narrative review to highlight clinical and research needs in the acute phase and the long return to normality. *Child and Adolescent Psychiatry and Mental Health*. 2020;14(1):20. doi: 10.1186/s13034-020-00329-3 32419840 PMC7216870

[23] Wardell JD, Kempe T, Rapinda KK, Single A, Bilevicius E, Frohlich JR, et al. Drinking to cope during COVID-19 pandemic: the role of external and internal factors in coping motive pathways to alcohol use, solitary drinking, and alcohol problems. *Alcoholism*, *Clinical and Experimental Research*. 2020;44(10):2073–83. Epub 2020/09/02. doi: 10.1111/acer.14425 32870516

[24] Dsouza DD, Quadros S, Hyderabadwala ZJ, Mamun MA. Aggregated COVID-19 suicide incidences in India: Fear of COVID-19 infection is the prominent causative factor. *Psychiatry Research*. 2020;290:113145. Epub 2020/06/17. doi: 10.1016/j.psychres.2020.113145 32544650 PMC7832713

[25] Syed NK, Griffiths MD. Nationwide suicides due to alcohol withdrawal symptoms during COVID-19 pandemic: a review of cases from media reports. *Journal of Psychiatry Research*. 2020;130:289–91. Epub 2020/09/01. doi: 10.1016/j.jpsychires.2020.08.021 32866677 PMC7438040

[26] Africanews. Schools remain closed in Uganda for over 77 weeks due to covid 19 2021. Available from: https://www.africanews.com/2021/10/28/schools-remain-closed-in-uganda-for-over-77-weeks-due-to-covid-19//.

[27] Weatherson KA, O’Neill M, Lau EY, Qian W, Leatherdale ST, Faulkner GEJ. The protective effects of school connectedness on substance use and physical activity. *The Journal of Adolescent Health*: *Official Publication of the Society for Adolescent Medicine*. 2018;63(6):724–31. Epub 2018/10/03. doi: 10.1016/j.jadohealth.2018.07.002 30269908

[28] Kabwama SN, Matovu JKB, Ssenkusu JM, Ssekamatte T, Wanyenze RK. Alcohol use and associated factors among adolescent boys and young men in Kampala, Uganda. *Substance Abuse Treatment*, *Prevention*, *and Policy*. 2021;16(1):49. doi: 10.1186/s13011-021-00385-8 34107981 PMC8191098

[29] World Health Organisation. Adolescent health 2021. Available from: https://www.who.int/health-topics/adolescent-health#tab=tab_1.

[30] The School Series. Kuber 2022. Available from: http://school-series.com/drugs/67-kuber#:~:text=Kuber%20is%20smokeless%20tobacco%20that,because%20it%20has%20no%20smell.

[31] Wabe NT. Chemistry, pharmacology, and toxicology of khat (catha edulis forsk): a review. Addiction & health. 2011;3(3–4):137–49. Epub 2011/07/01. 24494129 PMC3905534

[32] Kaggwa MM, Nkola R, Najjuka SM, Bongomin F, Ashaba S, Mamun MA. Extrapyramidal side effects in a patient with alcohol withdrawal symptoms: a reflection of quality of the mental health care system. *Risk Management and Healthcare Policy*. 2021;14:2789–95. doi: 10.2147/RMHP.S314451 34234593 PMC8257062

[33] Association of Maternal & Child Health Programs. Adolescent development 2021. Available from: http://www.amchp.org/programsandtopics/AdolescentHealth/projects/Pages/AdolescentDevelopment.aspx#:~:text=Researchers%20suggest%20adolescence%20undergo%20three,and%20late%20adolescence%2Fyoung%20adulthood.&text=Early%20Adolescence%20occurs%20between%20ages%2010%2D14.

[34] Basu D, Ghosh A. Substance use and other addictive disorders in international classification of Diseases-11, and their relationship with diagnostic and statistical Manual-5 and international classification of Diseases-10. *Indiaan Journal of Social Psychiatry*. 2018;34(5):54–62. doi: 10.4103/ijsp.ijsp_83_17

[35] World Medical Association. World Medical Association declaration of Helsinki: Ethical principles for medical research involving human subjects. *JAMA*. 2013;310(20):2191–4. doi: 10.1001/jama.2013.281053 24141714

[36] Miech R, Patrick ME, Keyes K, O’Malley PM, Johnston L. Adolescent drug use before and during U.S. national COVID-19 social distancing policies. *Drug and Alcohol Dependence*. 2021;226:108822. doi: 10.1016/j.drugalcdep.2021.108822 34214884 PMC8355118

[37] Dodge KA, Skinner AT, Godwin J, Bai Y, Lansford JE, Copeland WE, et al. Impact of the COVID-19 pandemic on substance use among adults without children, parents, and adolescents. *Addictive Behaviors Reports*. 2021;14:100388. doi: 10.1016/j.abrep.2021.100388 34938846 PMC8664966

[38] Sen LT, Siste K, Hanafi E, Murtani BJ, Christian H, Limawan AP, et al. Insights into adolescents’ substance use in a low–middle-income country during the COVID-19 pandemic. *Frontiers in Psychiatry*. 2021;12:1743. doi: 10.3389/fpsyt.2021.739698 34721110 PMC8551572

[39] Hearst MO, Fulkerson JA, Maldonado-Molina MM, Perry CL, Komro KA. Who needs liquor stores when parents will do? The importance of social sources of alcohol among young urban teens. *Preventive Medicine*. 2007;44(6):471–6. doi: 10.1016/j.ypmed.2007.02.018 17428525 PMC1987716

[40] Lenk KM, Toomey TL, Shi Q, Erickson DJ, Forster JL. Do sources of cigarettes among adolescents vary by age over time? *Journal of Child & Adolescent Substance Abuse*. 2014;23(2):137–43. Epub 2014/02/25. doi: 10.1080/1067828X.2012.750972 24563604 PMC3927921

[41] Argyriou E, Um M, Carron C, Cyders MA. Age and impulsive behavior in drug addiction: A review of past research and future directions. *Pharmacology*, *Biochemistry*, *and Behavior*. 2018;164:106–17. Epub 2017/08/06. doi: 10.1016/j.pbb.2017.07.013 28778737 PMC5797988

[42] Kasirye R. Uganda and the new alcohol control bill 2016 Sweden: Movendi International; 2016 [cited 2021]. Available from: https://movendi.ngo/blog/2016/09/26/uganda-new-alcohol-control-bill-2016/#:~:text=No%20person%20under%20the%20age,sale%20and%20consumption%20of%20alcohol.

[43] Dumbili EW. ’What a man can do, a woman can do better’: gendered alcohol consumption and (de)construction of social identity among young Nigerians. *BMC Public Health*. 2015;15:167. Epub 2015/04/18. doi: 10.1186/s12889-015-1499-6 25886193 PMC4340677

[44] Iwamoto DK, Cheng A, Lee CS, Takamatsu S, Gordon D. "Man-ing" up and getting drunk: the role of masculine norms, alcohol intoxication and alcohol-related problems among college men. *Addictive Behavoir* 2011;36(9):906–11. Epub 2011/05/31. doi: 10.1016/j.addbeh.2011.04.005 21620570 PMC3118921

[45] Wilsnack RW, Vogeltanz ND, Wilsnack SC, Harris TR, Ahlström S, Bondy S, et al. Gender differences in alcohol consumption and adverse drinking consequences: cross-cultural patterns. *Addiction (Abingdon*, *England)*. 2000;95(2):251–65. Epub 2000/03/21. doi: 10.1046/j.1360-0443.2000.95225112.x 10723854

[46] Moonajilin MS, Kamal MKI, Mamun FA, Safiq MB, Hosen I, Manzar MD, et al. Substance use behavior and its lifestyle-related risk factors in Bangladeshi high school-going adolescents: An exploratory study. *PLoS One*. 2021;16(7):e0254926. Epub 2021/07/22. doi: 10.1371/journal.pone.0254926 34288956 PMC8294555

[47] Meyer TD, McDonald JL, Douglas JL, Scott J. Do patients with bipolar disorder drink alcohol for different reasons when depressed, manic or euthymic? *Journal of Affective Disorders*. 2012;136(3):926–32. Epub 2011/10/05. doi: 10.1016/j.jad.2011.09.005 21967890

[48] Cetty L, Shahwan S, Satghare P, Devi F, Chua BY, Verma S, et al. Hazardous alcohol use in a sample of first episode psychosis patients in Singapore. *BMC Psychiatry*. 2019;19(1):91. doi: 10.1186/s12888-019-2073-z 30876474 PMC6419799

[49] Muhindo R, Joloba E. Health Management Information System (HMIS); Whose Data is it Anyway? Contextual Challenges. Review of Public Administration and Management. 2016;4. doi: 10.4172/2315-7844.1000190

