# Supplementary material for: Substance use disorder among adolescents before and during the COVID-19 pandemic in Uganda: Retrospective findings from a psychiatric ward registry
Source: PLoS One. 2022 May 26;17(5):e0269044. doi: 10.1371/journal.pone.0269044 (PMC9135190; doi:10.1371/journal.pone.0269044)
Supplement: Attachment — Submitted filename: Response to reviewers.docx [file pone.0269044.s001.docx]

PONE-D-22-08744

Substance use disorder among adolescents before and during the COVID-19 pandemic in Uganda: Retrospective findings from a psychiatric ward registry

PLOS ONE

**Response to the Reviewer 1’s Comment**

**Comment:** My only input is that I found the title of the manuscript not elaborately representative; this study does not describe Ugandan adolescence, its more like a description of the adolescents who have mental disorders in Uganda.

**Response:** We are thankful to the reviewer for their time to read and understand our manuscript. This study was a snapshot of adolescents diagnosed with substance use disorder during the COVID-19 pandemic and not necessarily those who had an only mental illness. This is also reflected in our study findings; that is, only five adolescents had a comorbid mental illness (i.e., depression, schizophrenia, etc.). Many adolescents used substances of addiction, but the study was interested in capturing those who were admitted due to complications of the substance use, i.e., substance use disorders.
